# Do infants fed directly from the breast have improved appetite regulation and slower growth during early childhood compared with infants fed from a bottle?

**DOI:** 10.1186/1479-5868-8-89

**Published:** 2011-08-17

**Authors:** Katherine I DiSantis, Bradley N Collins, Jennifer O Fisher, Adam Davey

**Affiliations:** 1 University of Pennsylvania, Perelman School of Medicine, Center for Clinical Epidemiology and Biostatistics, Philadelphia, PA, USA; 2 Temple University, College of Health Professions and Social Work, Department of Public Health, Philadelphia, PA, USA

**Keywords:** bottle-feeding, direct breastfeeding, satiety, obesity, child eating behaviors

## Abstract

**Background:**

Behavioral mechanisms that contribute to the association between breastfeeding and reduced obesity risk are poorly understood. The purpose of this study was to evaluate the hypothesis that feeding human milk from the breast (direct breastfeeding) has a more optimal association with subsequent child appetite regulation behaviors and growth, when compared to bottle-feeding.

**Methods:**

Children (n = 109) aged 3- to 6- years were retrospectively classified as directly breastfed (fed exclusively at the breast), bottle-fed human milk, or bottle-fed formula in the first three months of life. Young children's appetite regulation was examined by measuring three constructs (satiety response, food responsiveness, enjoyment of food) associated with obesity risk, using the Child Eating Behavior Questionnaire. Multinomial logistic regression analyses were used to test whether children bottle-fed either human milk or formula had reduced odds of high satiety and increased odds of high food responsiveness and high enjoyment of food compared to children fed directly from the breast. Current child weight status and growth trends from 6-36 months were also examined for their relation to direct breastfeeding and appetite regulation behaviors in early childhood.

**Results:**

Children fed human milk in a bottle were 67% less likely to have high satiety responsiveness compared to directly breastfed children, after controlling for child age, child weight status, maternal race/ethnicity, and maternal education. There was no association of bottle-feeding (either human milk or formula) with young children's food responsiveness and enjoyment of food. There was neither an association of direct breastfeeding with current child weight status, nor was there a clear difference between directly breastfed and bottle-fed children in growth trajectories from 6- to 36-months. More rapid infant changes in weight-for-age score were associated with lower satiety responsiveness, higher food responsiveness and higher enjoyment of food in later childhood

**Conclusion:**

While direct breastfeeding was not found to differentially affect growth trajectories from infancy to childhood compared to bottle-feeding, results suggest direct breastfeeding during early infancy is associated with greater appetite regulation later in childhood. A better understanding of such behavioral distinctions between direct breastfeeding and bottle-feeding may identify new pathways to reduce the pediatric obesity epidemic.

## Background

Obesity is a global problem even in the youngest populations. In the U.S., most recent reports indicate over 10% of infants and toddlers are overweight [1] and internationally, it is estimated that at least 20 million children under the age of 5 are overweight [2]. Increasing attention has been given to potential prevention strategies in early life [2]. Existing evidence indicates a protective effect of breastfeeding, accounting for as much as a 10-30% reduction in obesity risk through adulthood [3-7]. However, the mechanisms that explain this reduction in obesity risk are not fully understood.

Potential biological explanations of the breastfeed-obesity association have centered on differences in the nutritional composition of human milk versus formula, focusing in particular on the comparisons of human milk and formula in terms of protein and fatty acid composition [8]. For example, differences in the ratio of omega 3 and omega 6 fatty acids between human milk and formula have been associated with adipose tissue growth in infants [9] and insulin response in animal models [10]. A causal role for milk composition in promoting or protecting against obesity beyond infancy, however, has yet to be established [11].

Behavioral factors may explain the breastfeeding-obesity association, but these factors have not been fully explored. Both maternal and infant behaviors differ based on whether an infant is fed from the breast [direct breastfeeding] or bottle-fed human milk and/or formula. It is suggested that direct breastfeeding supports the development of appetite regulation given its inherent dependence on infant-centered feeding behaviors by the mother [12,13]. This process relies heavily on the infant's response to satiation rather than on visual cues, as a breastfeeding mother does not know how much milk is being offered, how much the infant is taking in, and also knows little about the rate with which their infant is drinking. Thus, direct breastfeeding requires the mother to focus on infant cues to gauge the child's interest in feeding (e.g. *increased alertness to caregiver, moving head towards caregiver*) and satiety *(e.g. reduced sucking, drowsiness, relaxed state, arms close to the body)*. In contrast, bottle-feeding, involving either human milk or formula, provides explicit visual information about infant intake to the caregiver based on the amount of milk or formula remaining in the bottle.

Limited empirical evidence exists to support the suggestion that direct breastfeeding is more infant-centered. To date, observational research examining mother-infant pairs at 1 week, 1 month, and 2 months of age has revealed that mothers who bottle-fed initiated a greater proportion of breaks during feeding than directly breast-feeding mothers [12]. Directly breastfed infants also exhibited greater variability in the volume of feeds, taking larger volume feedings following longer periods without milk/formula [12]. These findings suggest that direct breastfeeding may engender relatively less maternal control during feeding and more responsiveness to infant feeding cues than bottle-feeding. Lower levels of maternal control during feeding have been associated with less rapid infant growth during the first year of life [14] and lower Body Mass Index [BMI] z-scores (set to US reference population) at twelve months [15].

Although this evidence collectively suggests that the protective effect of breastfeeding on obesity risk may be partially driven by behavior, potential differences in the long-term appetite regulation between directly breastfed and bottle-fed infants has not been explored. Such an investigation would provide insight into the behavioral aspects of breastfeeding which might be related to weight gain and obesity. Identifying behavioral aspects unique to direct breastfeeding offers a potential path to child obesity prevention in the earliest years of life, as this identification could lead to interventions for all caregivers, including mothers who cannot or choose not to directly breastfeed (e.g. mother whose infant does not latch, mother who returns to work, mother who chooses to bottle-feed).

Reliance on internal cues during eating is important as there is a well-established relationship between appetite regulation and weight status [16-18]. Parental reports of poor use of internal cues (low satiety response) and increased use of external cues (high food responsiveness and high enjoyment of food) in children have been associated with increased adiposity and obesity, and are predictive of higher energy intake levels [19-21]. Whether behavioral aspects of infant feeding in the first year of life influence growth patterns later in childhood via appetite regulation has not been evaluated in detail.

Therefore, the primary aim of this study was to determine the association of direct breastfeeding during early infancy with later appetite regulation behaviors among 3- to 6-year-old children. Given associations with child obesity and energy intake, three aspects of child appetite regulation were examined: satiety response, food responsiveness, and enjoyment of food [19]. It was hypothesized that compared to children who were directly breastfed exclusively during the first three months of life [DIRECT BF], children who were bottle-fed either human milk [BTL-HM] or formula [BTL-FORM] would be less likely to display appetite regulation at 3-6 years old. As a secondary aim, it was hypothesized that direct breastfeeding would be associated with normal weight status and less rapid growth from infancy through early childhood compared to the BTL-HM and BTL-FORM children and that less rapid growth would in turn relate to child appetite regulation.

## Methods

### Design

This study employed a retrospective cohort design. Infant feeding group assignment was based on self-reported breastfeeding/human milk use and bottle-feeding behaviors during the first three months of life, which were verified by medical chart review. Current demographics, child appetite regulation behaviors, and anthropometric data were collected when children were 3-6 years old. Retrospective child growth data were assessed via medical records.

### Participants & Procedures

A sample of mothers and their 3-6 year old children were recruited using two strategies. The majority of recruitment took place in-person in the waiting area of a private pediatric primary care office located in suburban Philadelphia. A secondary method involved emails sent to members of a listserv provided by a non-profit, community-based breastfeeding center in the same geographic area. Breastfed and non-breastfed children were included in the study. Exclusion criteria included maternal history and/or current diagnosis of an eating disorder, maternal conditions that contraindicated breastfeeding (e.g. certain pharmaceutical use, double mastectomy), and a child being a multiple due to unique challenges related to breastfeeding higher order multiples [22].

After obtaining informed consent, participants completed the self-report surveys which took approximately 15 minutes. Those recruited in-person were asked to complete the survey during their pediatric clinic visit; those who did not complete it during the visit were asked to return the survey by prepaid mail provided by the researcher. Email-recruited participants received and returned consent forms and the survey packets by prepaid mail. A medical chart review of infant feeding history and child growth was performed for participant's recruited in-person from the pediatrician's office and for those recruited by e-mail who used the recruitment site pediatrician. For children with other pediatricians, mothers were asked to take the child growth forms to their pediatrician's office for completion, but infant feeding history was not verified for these participants. All procedures were carried out in accordance with Temple University human subjects' protections and U.S. privacy regulations regarding individually identifiable health information (HIPAA).

### Measures

#### Bottle Use & Feeding History

Duration of human milk use and bottle use (first three months of life) were self-reported by mothers. These data were verified for 78% of the sample (n = 85) using medical chart review (in regard to duration, exclusivity, and bottle use). Of the 85 charts reviewed, 91.6% were consistent with maternal self-report. Because five mothers overestimated and three mothers underestimated their length of exclusive human milk use, medical chart data were used in analyses for these participants. In order to isolate direct breastfeeding, mothers had to report exclusive direct breastfeeding (no bottle use) during the first three months of life for their children to be considered directly breastfed (DIRECT BF group). The reason for selecting the three month cut-off was twofold. First, most employed US women return to work within 12 weeks of giving birth and direct breastfeeding is less likely after this point [23]. Second, while the US recommendation is to use human milk or formula only during the first six months of life, most infants begin to transition to a mixed diet of breast milk, infant formula, and solid, spoon feeding (e.g. infant cereal) at 4 to 5 months of age [24]. Understanding these contextual factors that occur in the first 6 months, the first three months were focused on because direct breastfeeding, as a behavior, was expected to be at its greatest frequency and thus the behavioral differences between direct breastfeeding and bottle-feeding would be most pronounced.

Bottle use was determined based on a scaled response question, asking mothers who reported breastfeeding whether they typically breastfed from the breast in the first three months of life, "All of the time" (ex: never used a bottle on average days), "Most of the time" *(*ex: 0-1 bottles on average days), "Some of the time" (ex: 2-3 bottles on most days), and "None of the time" (ex: 4 or more bottles on most days). If a mother did not initiate breastfeeding, she was coded as feeding from the breast, "None of the time". Based on these responses, the DIRECT BF group was comprised of children who were fed human milk exclusively for the first three months of life and whose mothers stated they fed the infant at the breast all of the time. The BTL-HM group was compromised of children who were fed human milk (non-exclusive included) and whose mothers stated they fed the infant at the breast from most to none of the time during the first three months of life. The BTL-FORM group was comprised of children who were fed formula for the first three months of life. Four breastfed children were placed in the BTL-FORM group because they were supplemented with formula from birth and because, while mother's reported that they initiated breastfeeding, they said human milk was rarely fed due to breastfeeding difficulties (e.g. separation from infant at birth, insufficient milk supply, infant never latched). These breastfed children in the BTL-FORM group were all fed human milk for less than 4 weeks.

#### Child Regulation of Appetite

Appetite regulation was measured using selected subscales (Satiety Response [SR], Food Responsiveness [FR], and Enjoyment of Food [EF]) from the Child Eating Behavior Questionnaire [CEBQ], a 35-item parent-report measure of eight dimensions of young children's eating behaviors [19,25]. The SR subscale measures response to fullness while eating or choosing not to eat when one is full and it included statements like: "My child leaves food on his/her plate at the end of a meal." The FR subscale measures maladaptive eating which is normally triggered by external cues and it included statements like: "Even if my child is full, s/he finds room for his/her favorite food." The EF subscale measures general appetite and interest in food and it included statements like: "My child is interested in food." While the term "enjoyment of food" would be perceived as a positive quality, it has been associated with increased adiposity and overweight [20,21,25,26], and therefore is considered risky appetite characteristic that could lead to obesity. All three subscales have demonstrated a high internal consistency (SR: α = .83, FR: α = .82, EF: α = 0.91) and high test-retest reliability (SR: r = 0.85, FR: r = 0.83, EF: r = 0.87) in samples of preschool aged children [25]. These subscales have also demonstrated criterion validity in predicting behavioral measures of appetite regulation among preschool aged children including eating in the absence of hunger and caloric compensation during meals following caloric pre-loads [19]. All items on the CEBQ used 5-point Likert scale response options (0 = never, 4 = always). To allow for logistic regression analyses, each factor was converted into a dichotomous variable using a median split. The subscale scores below the median score for a subscale were grouped into a "low category" and those at or above the median were grouped into the "high category".

#### Child Growth Measures

Child weight at birth, as well as height and weight measurements taken at pediatric well-child visits at 6 months, 12 months, 24 months, 36 months and most recent visit, were collected by medical chart review. Weight previously recorded in pounds and height in inches (utilizing standardized methods of the recruitment site pediatric office) was converted to kilograms and meters, respectively. For 24% of child participants, growth data were obtained from the medical records of other pediatrician offices; therefore, methods of those length/height and weight measurements are less clear. The percentage of complete weight and length/height measurements at each of time point ranged from 94% to 96%, with the 6 month data having the lowest percentage for measurements obtained. Weight-for-length *z *scores [WFL*z*] (6, 12 month), BMI *z*-scores [BMI*z*] (24, 36 month, current) and weight-for-age *z *scores [WFA*z*] (6, 12, 24, 36 month) were calculated based on 2000 Centers for Disease Prevention and Control [CDC] growth standards [27].

### Family Demographics and Maternal Characteristics

Demographic information was collected by maternal self-report including maternal education level, parity at birth of the child participant, household income level, and maternal race/ethnicity. Maternal BMI was calculated based on maternal self-report of current height and weight. If a mother reported a current or recent pregnancy at the time of the interview, she was asked to report her weight prior to the pregnancy, in order to calculate a more valid BMI.

### Analysis Plan

Descriptive variables were examined with the entire sample, and compared between the three feeding groups (DIRECT BF, BTL-HM, BTL-FORM), where DIRECT BF children served as the reference group. For the primary outcome, multinomial logistic regressions were used to assess whether the directly breastfed children differed from the bottle-fed children in appetite regulation (high SR, high FR, high EF) after adjusting for possible confounders. A hierarchical approach was utilized in order to first assess the simple relationship between infant feeding method and child appetite regulation, with adjustments for the potentially confounding effects of covariates on individual basis (some covariates were grouped due to their interrelationships). A large set of covariates was considered due to their relationship with child appetite behaviors in the previous research, including maternal BMI [28], race/ethnicity [29], maternal restraint level in her own eating [30], and child gender [25]. However, only child age [31], child weight status [20], maternal race/ethnicity [29], and maternal education level [32] were included in the final models as they were significantly correlated with child appetite regulation (p ≤ 0.05). The models first accounted child characteristics then maternal race, and lastly maternal education. SPSS^® ^version 16 statistical software was used to run the primary analyses.

Our secondary analyses used t-tests to assess potential differences in growth (WFL/BMI z-score) between the DIRECT BF, BTL-HM, and BTL-FORM groups, across 6-36 months of age. Two-stage growth models (TSGM) were then used to assess potential differences in within-subjects growth trajectories. A regression model was run for each child participant to estimate an intercept and slope for each child's linear growth pattern centered on the first growth point measurement, 6 months. Because anthropometric measures were age-standardized, growth trends were linear. A quadratic model was considered, but rejected because coefficients were approximately zero with little variation across individuals. Thus, a linear model was retained for all subsequent analyses. Separate models were run for each growth outcome (WFL*z*/BMI*z *and WFA*z*) with infant feeding group as the predictor, controlling for maternal race, maternal education, and maternal weight status; the latter was included because of its relationship with child weight status in this sample. Lastly, to assess whether growth patterns predicted later appetite regulation, models were run for each of the appetite regulation behaviors [SR, FR, EF] with the growth measures as an outcome (WFL*z*/BMI*z *and WFA*z*), controlling for feeding group [DIRECT BF, BTL-HM, BTL-FORM]. STATA^® ^version 11 statistical software was used to run TSGM analyses.

## Results & Discussion

### Sample

Table 1 presents a description of the sample. The majority of mothers were enrolled (n = 84; 77%) during in-person recruitment at the private pediatric clinic. The percentage of mothers who agreed to participate was 87.9%, with six mothers declining to participate and nine mothers found to be ineligible. Reasons for ineligibility included existing or prior serious child health problems that could have affected eating or growth (n = 3), mother's prior or current diagnosis of eating disorder (n = 4), adopted child with mother having no knowledge of infant feeding (n = 1), and mother's inability to breastfeed due to pharmaceutical drug use (n = 1). The final sample consisted of primarily white, non-Hispanic children who ranged from 3-6 years old, with the average age being 4.1 years. Child gender was evenly distributed in the full sample and between groups. About 25% of child participants were overweight or obese at the time of the interview, with no significant differences in weight status between the groups. Forty-two percent of mother participants were classified as overweight or obese, which is lower than the national prevalence (59.5%) for adult women (aged 20-39 years) [33]. Differences in maternal overweight (BMI≥25) between DIRECT BF (40%), BTL-HM (37.8%) and BTL-FORM group (61.9%) were not statistically significant (p > 0.05). Family income varied significantly between groups, with the DIRECT BF group having more participants in the greater than $100,000 income level, as compared to the BTL-HM and BTL-FORM groups (p ≤ 0.05).

**Table 1:** Demographics of sample and demographics by breastfeeding group

Demographics	All Participants n = 109	DIRECT BF n = 40	BTL-HM n = 47	BTL-FORM n = 22
Childs Age, mean (SD)	4.1 (1.0)	4.0 (1.0)	4.0 (1.0)	4.3 (1.1)

Weeks of Any Human Milk Use, mean (SD)*	37.9 (42.8)	73.2 (47.3)	25.3 (22.0)	0.1 (1.6)

Weeks of Exclusive Human Milk Use, mean (SD)*	12.6 (10.5)	22.0 (5.5)	10.3 (8.9)	0 (-)

Mom Overweight or Obese**†**, n (%)	46 (42.2%)	16 (40%)	17 (37.8%)	13 (61.9%)

Child Weight Status: Overweight or Obese**†**, n (%)	26 (23.8%)	8 (20.0%)	12 (29.3%)	7 (31.8%)

**Child Gender**				

Male, n (%)	53 (48.6%)	22 (55.0%)	19 (40.4%)	12 (54.5%)

Female, n (%)	56 (51.4%)	18 (45.0%)	28 (59.6%)	10 (45.5%)

**Mom Race Ethnicity**				

White, non-Hispanic, n (%)	92 (84.4%)	37 (92.5%)	36 (76.6%)	19 (86.4%)

Black, African American, n (%)	11 (10.1%)	1 (2.5%)	7 (14.9%)	3 (13.6%)

Other, n (%)	6 (5.5%)	2 (5.0%)	4 (8.5%)	0 (0%)

**Child Race Ethnicity**				

White, non-Hispanic, n (%)	93(85.3%)	37 (92.5%)	37 (78.7%)	19 (86.4%)

Black, African American, n (%)	9 (8.3%)	0 (0%)	6 (12.8%)	3 (13.6%)

Other, n (%)	7 (6.4%)	3 (7.5%)	4 (8.5%)	0 (0%)

**Parity**				

Multiparous at Interview†, n (%)	93 (92.1%)	36 (94.7%)	38 (92.7%)	19 (86.4%)

Primiparous at Child's Birth, n (%)	47 (43.5%)	18 (45.0%)	22 (47.8%)	7 (31.8%)

Household Income Level*				

Unknown, n (%)	5 (4.6%)	2 (5.2%)	3 (6.4%)	0 (0%)

< $50,000, n (%)	3 (2.8%)	1 (2.6%)	2 (4.2%)	0 (0%)

$50,000-$74,999, n (%)	10 (10.4%)	4 (10.5%)	3 (6.4%)	4 (18.2%)

$75,000-$99,999, n (%)	22 (20.8%)	2 (5.3%)	14 (29.8%)	6 (27.3%)

≥$100,000, n (%)	69 (66%)	31 (81.6%)	25 (53.2%)	12 (54.5%)

**Maternal Education Level**				

High School diploma or less, n (%)	16 (14.7%)	2 (5%)	10 (21.7%)	4 (18.2%)

College Graduate or Beyond, n (%)	93 (85.3%)	38 (95%)	37 (78.7%)	18 (81.8%)

†Some missing participant data- percentages based on complete data only.

*p ≤ 0.05

**p ≤ 0.10

Direct BF = direct breastfeeding group. BTL-HM = Bottle-fed Human Milk group. BTL-FORM = Bottle-fed Formula group.

The DIRECT BF group had a minimum of 12 weeks of exclusive direct breastfeeding with a mean of 73.2 weeks (*sd = *47.4) of any human milk use (range 16-200 weeks) and 22.0 weeks (*sd = *5.5) of exclusive human milk use. In the DIRECT BF group, 41.4% of women reported formula supplementation after the first three months of life and for these women the mean infant age of formula supplementation was 29.2 weeks (*sd = *10.9). Mean age of solid supplementation for the DIRECT BF group was 5.5 months (*sd = *1.2). The BTL-HM group had a mean of 25.3 weeks (*sd *= 22.0) of any human milk use and 10.3 weeks (*sd = *8.9) of exclusive human milk use. Mean age of solid supplementation for the BTL-HM group was 5.5 months (*sd = *1.4). For the BTL-FORM group, formula supplementation began at birth, with 0 weeks of exclusive human milk use. Four children (18.2%) in the BTL-FORM group were breastfed. The BTL-FORM group had a mean of 0.4 weeks (*sd = *1.1) of any human milk use, where mothers reported feeding from the breast "none of the time". The mean age for solids supplementation was 4.7 months (*sd = *1.0) in the BTL-FORM group.

### Association of Feeding Method and Child Appetite Regulation

**Figure 1:**
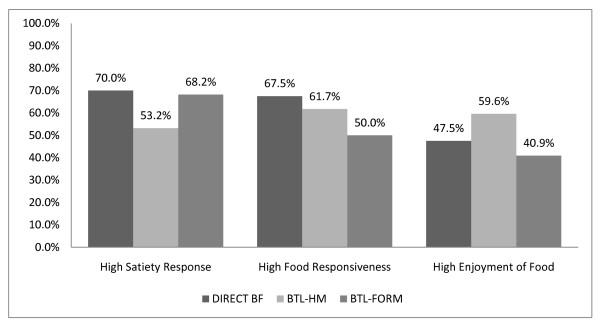
**The percentage of children in the high category for satiety responsiveness, food responsiveness, and enjoyment of food by group**.

SR, FR, and EF subscales demonstrated acceptable internal consistency in the current sample (SR: α = 0.72, FR:α = 0.77, EF: α = 0.87). On a 0-4 scale, the SR score median was 2.0, ranging from 0.6-4.0, with 36.9% having low vs. 63.1% high satiety response following median split, for the entire sample. The FR subscale score median was 1.3, ranging from 0.0-3.6, with 39.1% having low vs. 60.9% high food responsiveness following the median split. The EF subscale score median was 2.5, ranging from 0.75-4.0, with 48.2% sample having low vs. 51.8% having high enjoyment of food following the median split. Figure 1 presents the distribution of children in the high category for SR, FR, and EF subscales by group (DIRECT BF, BTL-HM, BTL-FORM).

Covariates were selected for inclusion in the multivariate model based on their correlation with child appetite regulation, thus child age at time of interview, child BMI, maternal race, and maternal education level were included in all analyses. Child age was negatively correlated with high SR (r = -0.22, p ≤ 0.05). Child BMI was negatively correlated with high SR (r = -0.25, p ≤ 0.01), low FR (r = -0.026, p ≤ 0.01), and low EF (r = -0.29, p ≤ 0.01). Using Chi-square analyses, it was found that 73% of children with low FR had mothers who were Black (χ^2 ^= 6.93, p ≤ 0.01). Using Chi-square analyses, it was found that 59% of children with high SR had mothers who were educated beyond the college degree level (χ^2 ^= 10.56, p ≤ 0.01).

Table 2 presents the odds ratio for high SR, FR, and EF for the BTL-HM and BTL-FORM groups when the DIRECT BF group is set as the reference population for multinomial logistic regression analyses. In the unadjusted model, the BTL-HM group did not have significantly reduced odds of high SR (OR: 0.49; 95% CI: 0.20, 1.18). However, after adjustment for child age and child weight status, the BTL-HM group had reduced odds of high SR (OR: 0.37; 95% CI: 0.14, 0.97). The final model, which included child age, child weight status, maternal race, and maternal education level, revealed the BTL-HM group was 67% less likely to have high satiety responsiveness as compared to the DIRECT BF group. The BTL-FORM group did not differ significantly from the DIRECT BF group in terms of odds of high satiety responsiveness, in the unadjusted or adjusted models.

**Table 2:** Crude and Adjusted Odds Ratio (OR) for appetite regulation at age 3-6 years for directly breastfed children compared to children bottle-fed human milk and formula

	High Satiety Responsiveness			High Food Responsiveness			High Enjoyment of Food		
	**DIRECT BF†** **OR (CI 95%)**	**BTL-HM** **OR (CI 95%)**	**BTL-FORM** **OR (CI 95%)**	**DIRECT BF†** **OR (CI 95%)**	**BTL-HM** **OR (CI 95%)**	**BTL-FORM** **OR (CI 95%)**	**DIRECT BF†** **OR (CI 95%)**	**BTL-HM** **OR (CI 95%)**	**BTL-FORM** **OR (CI 95%)**

Unadjusted	1.00	0.49 (0.20, 1.18)	0.92 (0.30, 2.82)	1.00	0.78 (0.32, 1.88)	0.48 (0.17, 1.40)	1.00	1.63 (0.70, 3.82)	0.77 (0.27, 1.40)

Model 1‡	1.00	0.37 (0.14, 0.97)*	1.24 (0.37, 4.14)	1.00	0.90 (0.35, 2.35)	0.42 (0.14, 1.25)	1.00	1.95 (0.79, 4.87)	0.61 (0.20, 2.19)

Model 2‡	1.00	0.34 (0.13, 0.93)*	1.21 (0.36, 4.04)	1.00	1.05 (0.39, 2.85)	0.45 (0.15, 1.35)	1.00	2.09 (0.81, 5.37)	0.33 (0.20, 1.89)

Model 3‡	1.00	0.33 (0.12, 0.93)*	1.18 (0.35, 3.99)	1.00	1.26 (0.45, 3.54)	0.48 (0.16, 1.48)	1.00	2.23 (0.85, 5.90)	0.64 (0.21, 1.95)

†DIRECT BF was Reference group.

‡ Model 1 includes child age and child weight status

Model 2 includes Model 1 + maternal race

Model 3 includes Model 2 + maternal education level

* p ≤ 0.05

DIRECT BF = direct breastfeeding group. BTL-HM = Bottle-fed Human Milk group. BTL-FORM = Bottle-fed Formula group. CI 95% denotes the 95% confidence interval. Child Weight Status was based on the current growth percentile, based on CDC standard growth reference, as follows: not overweight/obese as less than the 85th percentile for age and gender, and overweight/obese as equal to or greater than the 85th percentile for age and gender. Maternal Race was coded as being White or not. Maternal Education was coded as having a College degree and Beyond or having less than a College degree.

Table 2 also provides the odds ratio of high FR and high EF for the BTL-HM and BTL-FORM groups when the DIRECT BF group was set as the reference population. In the unadjusted model, the BTL-HM and BTL-FORM groups were not significantly more likely than the DIRECT BF group to have high FR or high EF at age 3-6 years. After adjustment for all covariates, the BTL-HM and BTL-FORM groups did not differ significantly from the DIRECT BF group in level of food responsiveness or enjoyment of food at age 3-6 years.

### Child Growth Trends related to Feeding Method and Child Appetitive Self-Regulation

#### Feeding Method and Child Growth

Direct breastfeeding during first three months of life was assessed as a predictor of weight status and growth. Table 3 presents WFL/BMI z-score means for the DIRECT BF, BTL-HM, and BTL-FORM groups at all time points (6 mo, 12 mo, 24 mo, 36 mo, Current). T-tests did not identify significant differences in these relative weight measures across the five time points between the groups. Direct breastfeeding (DIRECT BF vs. BTL-HM or BTL-FORM) did not predict growth patterns (neither intercept, slope) (see Table 4).

**Table 3:** Weight for Length and Body Mass Index z-scores Description by Feeding Group from 6 months to current

	DIRECT BF	BTL-HM	BTL-FORM
**Month 6**			

WFL*z *, Mean (sd)	0.68 (1.01)	0.61 (0.91)	0.83 (1.24)

**Month 12**			

WFL*z*, Mean (sd)	0.45 (1.15)	0.57(0.82)	0.58 (1.21)

**Month 24**			

WFL*z*, Mean (sd)	0.23 (1.17)	0.36 (1.15)	0.12 (1.32)

**Month 36**			

BMI*z*, Mean (sd)	0.36 (1.09)	0.27 (0.91)	0.16 (0.98)

**Current**			

***Mean Age at Measurement***	***4.00 (1.04)***	***4.00 (0.98)***	***4.20 (1.11)***

BMI*z*, Mean (sd)	0.48 (0.89)	0.35 (1.06)	0.46 (1.00)

Direct BF = direct breastfeeding group. BTL-HM = Bottle-fed Human Milk group. BTL-FORM = Bottle-fed Formula group. WFL*z *= Weight for Length z-score. WFL% = Weight-for-length percentile. BMIz = body mass index z-score. BMI% = body mass index percentile.

**Table 4:** Parameter Estimates of the Latent Growth Models of WFL*z*/BMI*z *and WFA*z *by group^a^

	BTL-HM		BTL-FORM	
	**Intercept, Coef. (Std. Err.)**	**Slope, Coef. (Std. Err.)**	**Intercept, Coef. (Std. Err.)**	**Slope, Coef. (Std. Err.)**

WFL*z*/BMI*z*	-0.17 (0.22)	0.04 (0.02)	-0.08 (0.27)	-0.01 (0.03)

WFA*z*	0.18 (0.23)	0.02 (0.02)	0.07 (0.28)	0.00 (0.02)

#### Child Growth and Child Appetitive Self-Regulation

Growth patterns (WFL*z*/BMI*z*, WFA*z*) were associated with appetite regulation (see Table 5). Children with a history of more rapid weight gain (WFA*z*) from 6-36 months were more likely to have lower SR at ages 3-6. Children with more rapid weight gain (WFA*z*) from 6-36 months were also more likely to have high FR at ages 3-6, but rapid weight gain was not a significant predictor of higher EF at ages 3-6. More rapid change in relative growth (WFL*z*/BMI*z*) from 6-36 months, was not a significant predictor of lower SR, higher FR, or higher EF.

**Table 5:** Parameter Estimates of the Latent Growth Models of CEBQ subscale scores by WFL*z*/BMI*z *and WFAz^b^

	Intercept, Coef. (Std. Err.)	Slope, Coef. (Std. Err.)
SR, WFL*z*/BMI*z*	-0.07 (0.05)	-0.91 (0.54)

SR, WFA*z*	-0.08 (0.06)	-1.33 (2.22)*

FR, WFL*z*/BMI*z*	0.14 (0.08)	1.02 (0.74)

FR, WFA*z*	0.62 (0.07)	1.93 (0.85)*

EF, WFL*z*/BMI*z*	0.04 (0.08)	-0.41 (0.81)

EF, WFA*z*	-0.02 (0.08)	0.68 (0.83)

* p ≤ 0.05

^a ^DIRECT BF was the reference group. Controlling for: Overall duration of human milk use, child gender, child race, maternal education, and income level

^b ^Controlling for: Group Membership [DIRECT BF, BTL-HM, BTL-FORM]

BTL-HM = Bottle-fed Human Milk group. BTL-FORM = Bottle-fed Formula group. BMIz = body mass index z-score. WFL*z *= Weight-for-Length z-score. WFA*z *= Weight-for-Age z-score. SR = Satiety Responsiveness Subscale. FR = Food Responsiveness Subscale. EF = Enjoyment of Food Subscale. Coef = Coefficient. Std. Err. = Standard Error. The total sample was n = 99 for these analyses.

### Discussion of Results

This study provides evidence that direct breastfeeding during early infancy is related to greater appetite regulation later in childhood. In this study, children who were fed human milk in a bottle during the first three months of life were 67% less likely to have high satiety responsiveness at the age of 3-6 years when compared to children who were directly breastfed after considering child age, child weight status, maternal race, and maternal education level. Children fed formula in a bottle did not differ significantly from children who were directly breastfed in terms of likelihood of appetite regulation; however, the sample size of formula-fed children was small (n = 22) which might have limited our ability to identify differences. In parallel to previous research which connects parental feeding practices (e.g. control) and child appetite regulation [34,35], our findings suggest that direct breastfeeding may have lasting effects on children's appetite regulation.

Examining the potential connection of direct breastfeeding and later satiety response is novel. Our results suggest a potential utility of studying maternal-infant interactions and behaviors surrounding breastfeeding in order to understand the development of appetite regulation, beyond observing differences based on milk type alone. Breastfeeding duration can assess the overall length of exposure to and/or dosage of human milk, but feeding human milk directly from the breast versus milk from a bottle reflects potential behavioral differences in feeding which may shape infant consumption patterns. Bottle-feeding provides visual cues to mothers/caregivers about the volume of milk consumed, which might encourage a caregiver to feed and/or an infant to eat independent of internal hunger and satiety cues [12,36]. A chronic pattern of continuing to feed an infant after satiety cues are exhibited may increase children's subsequent responsiveness to external food cues (including caregiver prompts) and risk of overeating [37]. Recent research on infant bottle emptying (used as an indicator of low infant self-regulation) supports this assertion, revealing that infants who were directly breastfed from 0-6 months empty bottles less often in later infancy (27% of the time) compared to infants bottle-fed either human milk (54%) or formula (68%) [38].

The current study evaluated the effect of direct breastfeeding on overweight or less rapid growth from 6-36 months, finding no relation to either. While past research finds that longer breastfeeding duration is protective obesity throughout childhood [6,39], this study assessed the effects of direct breastfeeding which is why might diverge from some of the breastfeeding versus formula literature. The only other study which has assessed the effects of direct breastfeeding on infant self-regulation did not assess weight outcomes [38], thus there is not literature on which these results can be compared. The child overweight/obesity rates of this sample were similar to the U.S. population, therefore it was expected that the data would support this hypothesis. In trying to understand why this might be, it was considered that this study had little information about important child characteristics which affect weight status and growth patterns including diet quality [40], physical activity levels [41], and screen time [42]. Also, the familial and home environment, which might provide resources to modify the effect of lower satiety response (e.g. parental knowledge in healthy eating behaviors, high nutrition-low calorie food availability), was not assessed here. Therefore, a more comprehensive exploration is needed in order to understand whether direct breastfeeding can reduce obesity risk via improved satiety response. Future studies should include populations at higher risk for obesity, including children from families of low-income and lower parental education level [43] and African American and Hispanic children [1], as direct breastfeeding might have differential effects on more high-risk children.

Child appetite regulation at ages 3-6 years was related to weight gain from 6 months to 3 years, but was not related to change in relative growth (WFL*z*/BMI*z*). Lower satiety response was associated with a history of rapid weight gain from infancy through childhood, whereas higher food responsiveness was related to history of rapid growth. Because appetite regulation assessments followed growth measurements, causality cannot be discerned. These findings advance previous knowledge on these constructs which has demonstrated links to weight, but only in cross-sectional fashion [20,21,26]. Past findings and those of the current study emphasize the importance of longitudinal investigation of feeding effects on child development, in order to further understand whether appetitive regulation is related to the trajectory of growth in early childhood.

### Limitations

Several limitations of the current study must be considered when interpreting these results. In regards to the generalizability of the findings, this sample was primarily non-Hispanic white, highly educated, and of higher income, thus to the extent to which the findings generalize to other racial, educational, and income groups is unclear. Recall of feeding method (bottle use, duration of human milk use) was a limitation; however, the use of chart review provided evidence that mothers' retrospective reports were reasonably accurate reflections of medical chart notes. Maternal reports of child appetite regulation, assessed by questionnaire, have been previously associated with obesity in children [19]. However, a richer dataset would include observational data on child appetite regulatory behaviors. Lastly, because the current study accessed a population with high breastfeeding rates, there were few children in the sample who were fed formula-only from birth, which reduced the power of comparisons between children who were directly breastfed and children who were primarily fed formula in a bottle from birth. Future studies should seek to utilize larger and more diverse samples in order to further understand the unique contribution of direct breastfeeding to appetite regulation.

## Conclusions

This investigation yields new evidence of an association between direct breastfeeding and children's satiety response during early childhood. The findings point to differences in the behavioral processes surrounding feeding method, suggesting that feeding human milk from the breast may support the development of satiety responsiveness across infancy and childhood. Additional research is needed to understand whether the infant-centered experience of direct breastfeeding influences how mothers approach feeding beyond weaning. Such information is needed to promote optimal feeding interactions for all caregivers including mothers who wean, mothers who cannot or choose not to breastfeed, and other caregivers (e.g. fathers, grandparents, and non-relative caregivers). Through further inquiry and interventions, our understanding of behavioral factors which are distinct to direct breastfeeding could be improved, which in turn could accelerate and enhance our efforts to reduce the pediatric obesity epidemic

## Abbreviation List

BMI: Body Mass Index; BTL-FORM: Bottle-fed Formula group; BTL-HM: Bottle-fed Human Milk group; DIRECT BF: Directly Breastfed group; SR: Satiety Responsiveness; FR: Food Responsiveness; EF: Enjoyment of Food; CEBQ: Child Eating Behavior Questionnaire; HIPPA: Health Insurance Portability and Accountability Act; WFLz: Weight-for-Length z-score; BMIz: Body Mass Index z-score; WFAz: Weight-for-age z-score; TSGM: Two Stage Growth Model; CDC: Centers for Disease Prevention and Control

## Competing interests Statement

The authors declare that they have no competing interests.

## Authors' contributions

Significant writer (KID, AD), significant reviewer (BNC and JOF), manuscript concept/design (KID, BNC, and JOF), data acquisition (KID), data analysis (KID, AD), and statistical expertise (AD). All authors have read and approved the final manuscript.
